# Supplementary material for: Brain Targeting by Intranasal Drug Delivery: Effect of Different Formulations of the Biflavone “Cupressuflavone” from Juniperus sabina L. on the Motor Activity of Rats
Source: Molecules. 2023 Jan 31;28(3):1354. doi: 10.3390/molecules28031354 (PMC9921169; doi:10.3390/molecules28031354)
Supplement: Supplementary file 1 [file molecules-28-01354-s001.zip › molecules-2100355-supplementary.pdf]

molecules

Article

Brain Targeting by Intranasal Drug Delivery: Effect of Different Formulations of the Biflavone "Cupressuflavone" from *Juniperus Sabina* L. on the Motor Activity of Rats

El-Sayed Khafagy [1,2,*], Gamal A. Soliman [3,4], Ahmad Abdul-Wahhab Shahba [5], Mohammed F. Aldawsari [1], Khalid M. Alharthy [3], Maged S. Abdel-Kader [6,7] and Hala H. Zaatout [7]

1 Department of Pharmaceutics, College of Pharmacy, Prince Sattam bin Abdulaziz University, Al-kharj 11942, Saudi Arabia
2 Department of Pharmaceutics and Industrial Pharmacy, Faculty of Pharmacy, Suez Canal University, Ismailia 41522, Egypt
3 Department of Pharmacology, College of Pharmacy, Prince Sattam Bin Abdulaziz University, P.O. Box 173, Al-Kharj 11942, Saudi Arabia
4 Department of Pharmacology, College of Veterinary Medicine, Cairo University, Giza 12211, Egypt
5 Kayyali Chair for Pharmaceutical Industries, Department of Pharmaceutics, College of Pharmacy, King Saud University, P.O. Box 2457, Riyadh 11451, Saudi Arabia
6 Department of Pharmacognosy, College of Pharmacy, Prince Sattam Bin Abdulaziz University, P.O. Box 173, Al-Kharj 11942, Saudi Arabia
7 Department of Pharmacognosy, Faculty of Pharmacy, Alexandria University, Alexandria 21215, Egypt
* Correspondence: e.khafagy@psau.edu.sa

Table of contents

Title	Page
**Table S1.** ^{1}H in ppm (multiplicity, *J* in parentheses in Hz) of **1**-3 **4** in CD_3OD ……	3
**Table S2.** ^{13}C-NMR data in ppm of **1**, **2** and **4** in CD_3OD ……	3
**Figure S1.** ^{1}HNMR spectrum of **1**……	4
**Figure S2.** ^{1}HNMR spectrum of **1 (Exp.)**……	4
**Figure S3.** ^{13}CNMR spectrum of **1.** ……	5
**Figure S4.** DEPT135 spectrum of **1** ……	5
**Figure S5.** HSQC spectrum of **1.** ……	6
**Figure S6.** HRESIMS spectrum of **1.** ……	6
**Figure S7.** ^{1}HNMR spectrum of **2.** ……	7
**Figure S8.** ^{1}HNMR spectrum of **2 (Exp.).** ……	7
**Figure S9.** ^{13}CNMR spectrum of **2.** ……	8
**Figure S10.** DEPT135 spectrum of **2.** ……	8
**Figure S11.** COSY spectrum of **2.** ……	9
**Figure S12.** HSQC spectrum of **2** ……	9
**Figure S13.** HSQC spectrum of **2 (Exp.).** ……	10
**Figure S14.** HRESIMS spectrum of **2.** ……	10
**Figure S15.** ^{1}HNMR spectrum of **3.** ……	11
**Figure S16.** ^{1}HNMR spectrum of **3 (Exp.).** ……	11
**Figure S17.** ^{13}CNMR spectrum of **3.** ……	12
**Figure S18.** ^{13}CNMR spectrum of **3 (Exp.)** ……	12
**Figure S19.** ^{13}CNMR spectrum of **3 (Exp.)** ……	13
**Figure S20.** DEPT135 spectrum of **3** . ……	13
**Figure S21.** COSY spectrum of **3.** ……	14
**Figure S22.** HSQC spectrum of **3** ……	14
**Figure S23.** HSQC spectrum of **3 (Exp.).** ……	15
**Figure S24.** HRESIMS spectrum of **3.** ……	15

Table S1. ^{1}H-NMR in ppm (multiplicity, *J* in parentheses in Hz) of **1**-3 in CD_3OD.

	**1-I**	**2-I**	**3-I**		**1-II**	**2-II**	**3-II**
**3**	6.81, s	6.76, s	6.56, s	**3**	6.81, s	6.67, s	6.38, s
**6**	6.49 s	5.98 s	-	**6**	6.49 s	6.15 s	6.17 (bs)
**8**	-	-	5.83, s	**8**	-	6.27 s	6.05 (bs)
**2′**	7.52 (d, *J*= 8.8)	7.69 (d, *J*= 8.6)	7.83 (d, *J*= 7.6)	**2′**	7.52 (d, *J*= 8.8)	8.32 (d, *J*= 1.8)	8.18 (bs)
**3′**	6.76 (d, *J*= 8.8)	6.53 (d, *J*= 8.6)	6.87 (d, *J*= 7.6)	**3′**	6.76 (d, *J*= 8.8)	-	-
**5′**	6.76 (d, *J*= 8.8)	6.53 (d, *J*= 8.6)	6.87 (d, *J*= 7.6)	**5′**	6.76 (d, *J*= 8.8)	6.86 (d, *J*= 8.6)	6.79 (d, *J*= 8.3)
**6′**	7.52 (d, *J*= 8.8)	7.69 (d, *J*= 8.6)	7.83 (d, *J*= 7.6)	**6′**	7.52 (d, *J*= 8.8)	7.87 (dd, *J*= 1.8, 8.6	7.66 (bd, *J*=8.3)

Table S2. ^{13}C-NMR data in ppm of **1**-3 in CD_3OD.

	**1-I**	**2-I**	**3-I**		**1-II**	**2-II**	**3-II**
**2**	163.0	165.1	163.9	**2**	163.0	165.1	164.2
**3**	103.0	102.2	101.6	**3**	103.0	102.8	101.5
**4**	182.6	182.5	181.4	**4**	182.6	182.2	180.6
**5**	161.5	160.9	159.5	**5**	161.5	161.4	161.6
**6**	99.0	102.6	103.2	**6**	99.0	99.2	97.1
**7**	161.3	161.4	163.0	**7**	161.3	160.9	164.7
**8**	98.9	107.8	101.4	**8**	98.9	94.4	95.7
**9**	155.3	155.2	157.3	**9**	155.3	157.7	158.5
**10**	104.2	101.9	100.5	**10**	104.2	103.8	100.7
**1′**	121.6	122.1	124.9	**1′**	121.6	117.9	112.7
**2′**	128.4	128.5	128.4	**2′**	128.4	131.8	131.6
**3′**	116.3	115.9	117.0	**3′**	116.3	124.3	118.6
**4′**	164.1	164.5	161.6	**4′**	164.1	160.8	158.5
**5′**	116.3	115.9	117.0	**5′**	116.3	120.20	119.9
**6′**	128.4	128.5	128.4	**6′**	128.4	126.70	125.7

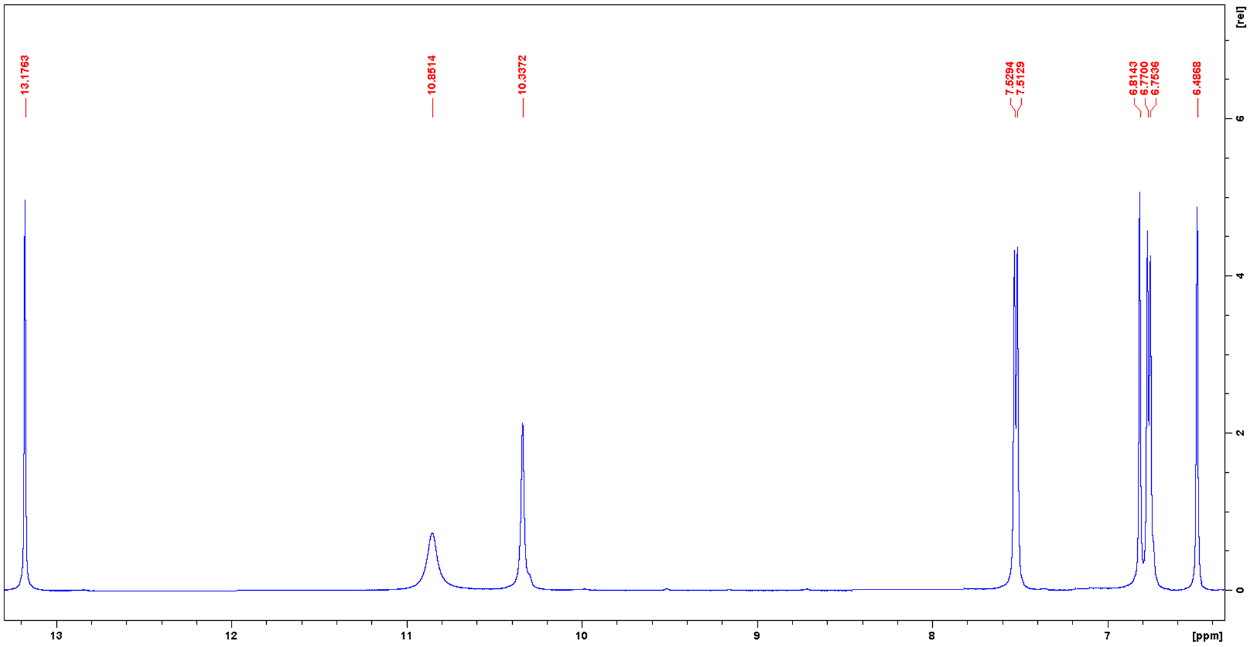

Figure S1: ^{1}HNMR of **1**.

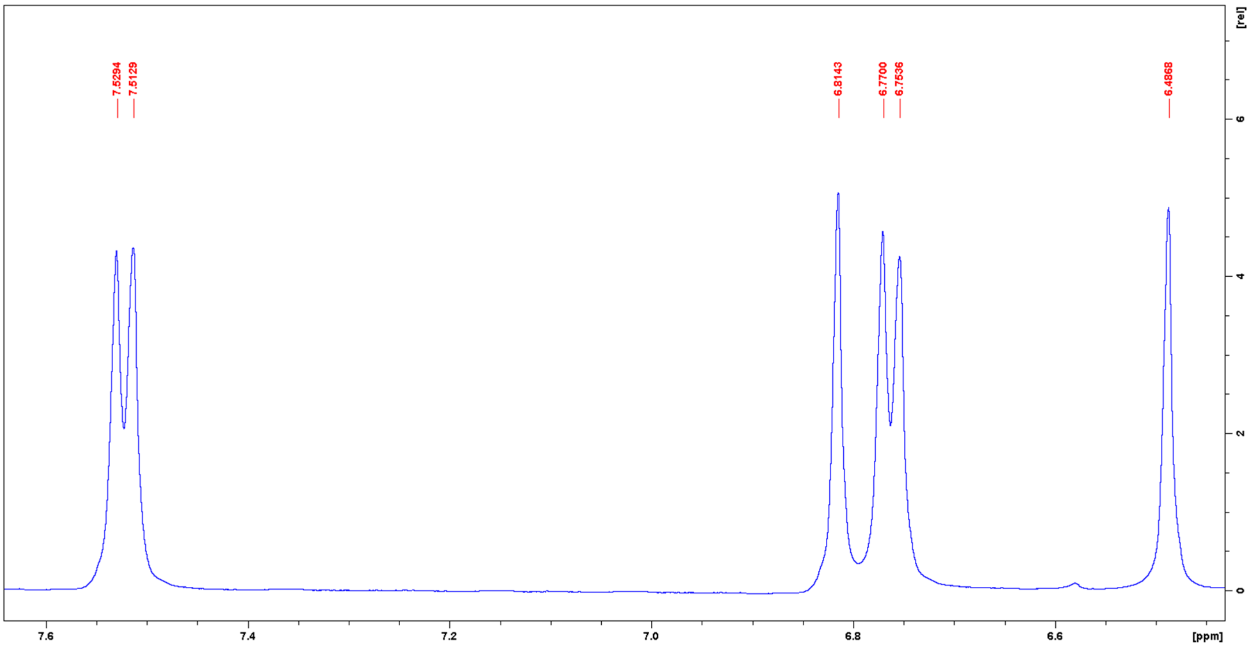

Figure S2: ^{1}HNMR of **1** (Exp.).

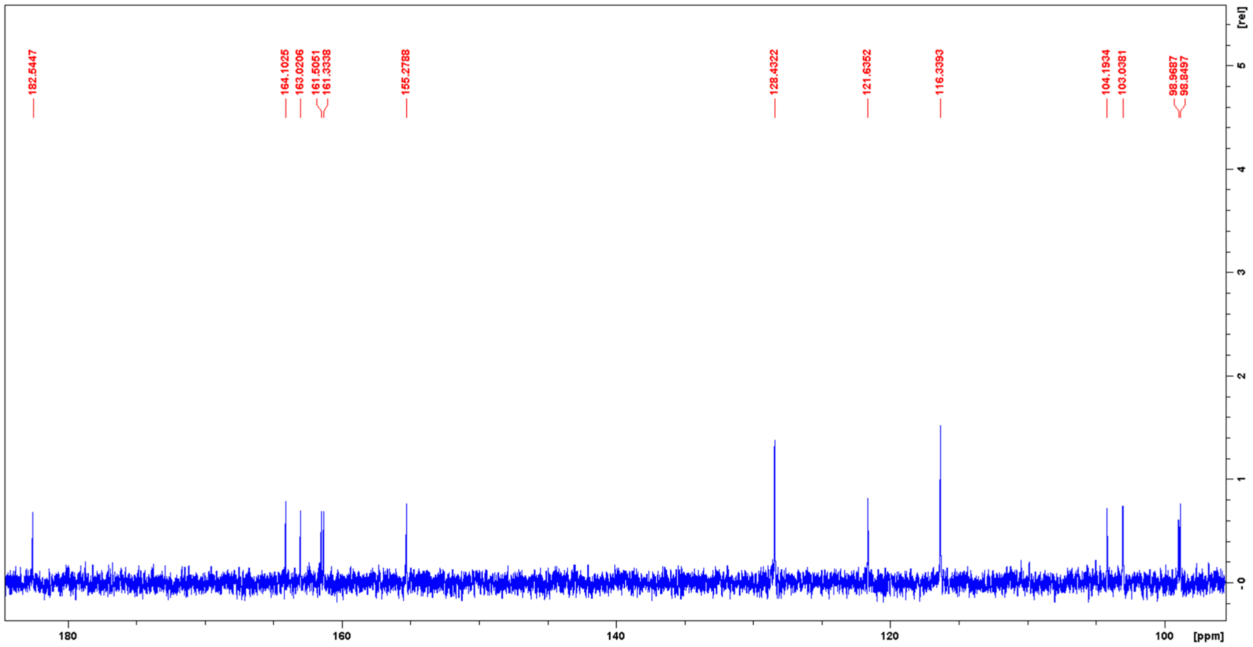

Figure S3: ^{13}CNMR of **1**.

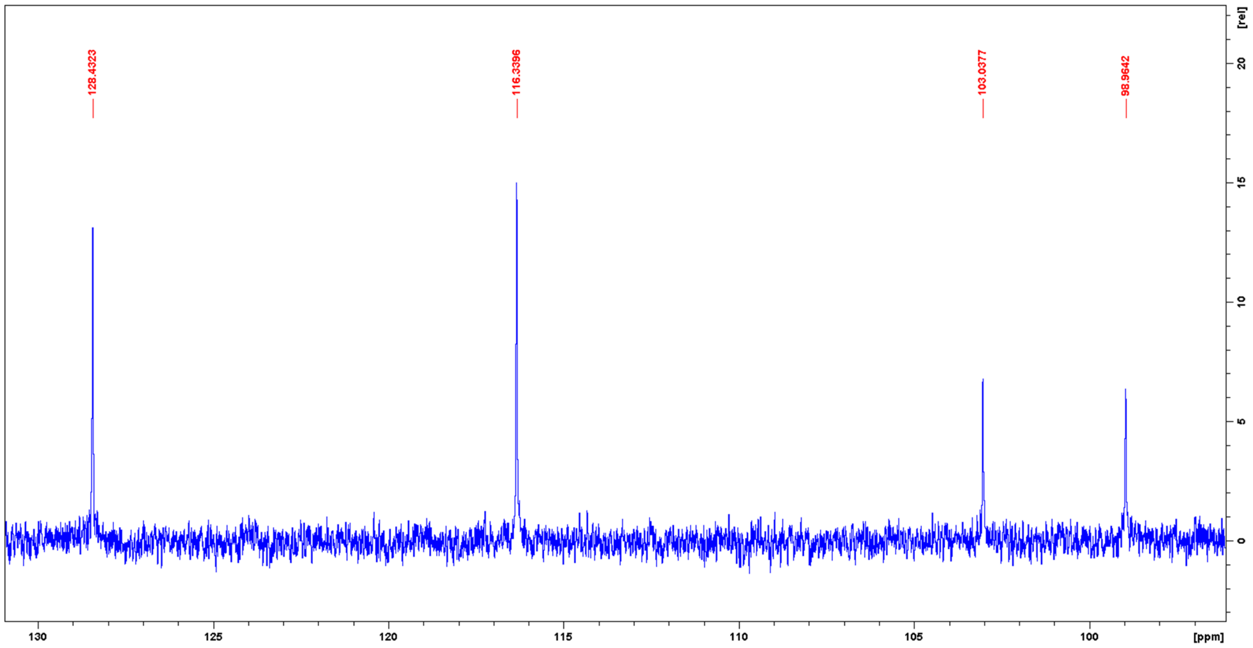

Figure S4: DEPT135 of **1**.

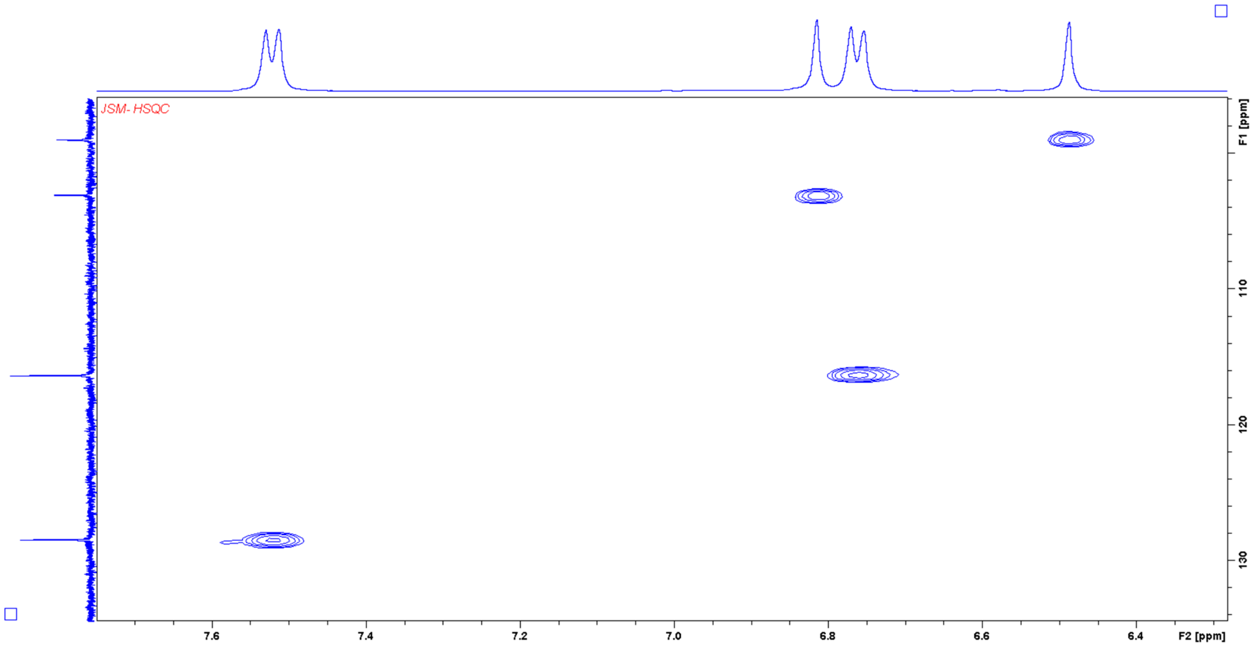

Figure S5: HSQC of **1**.

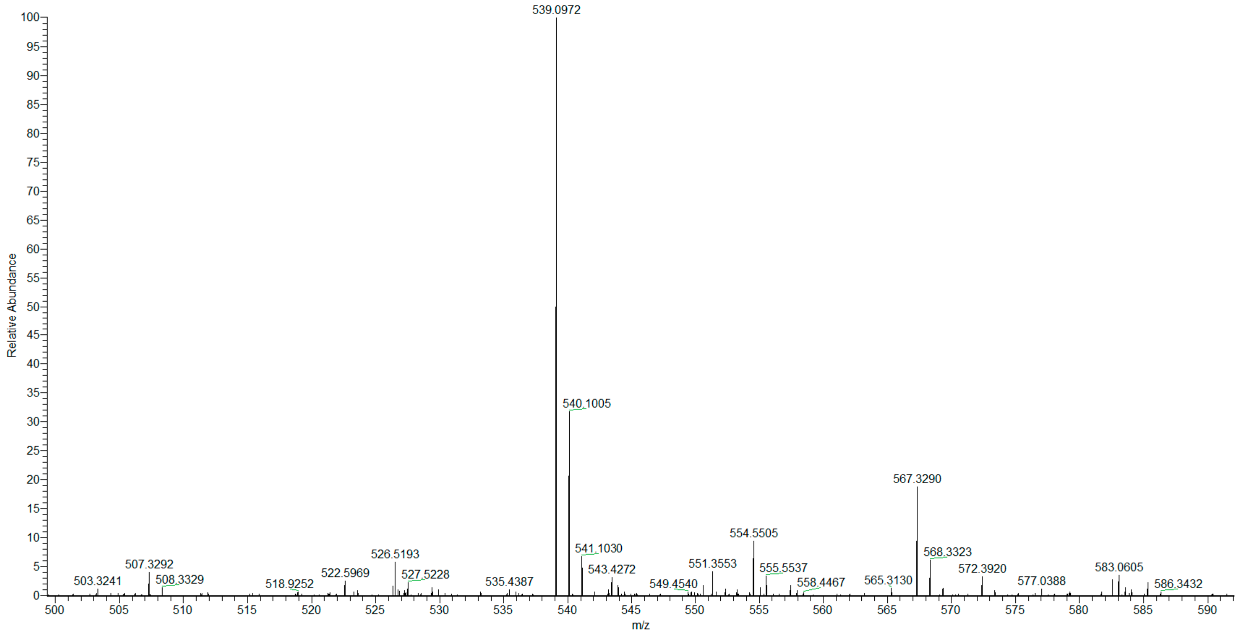

Figure S6: HRESIMS of **1**.

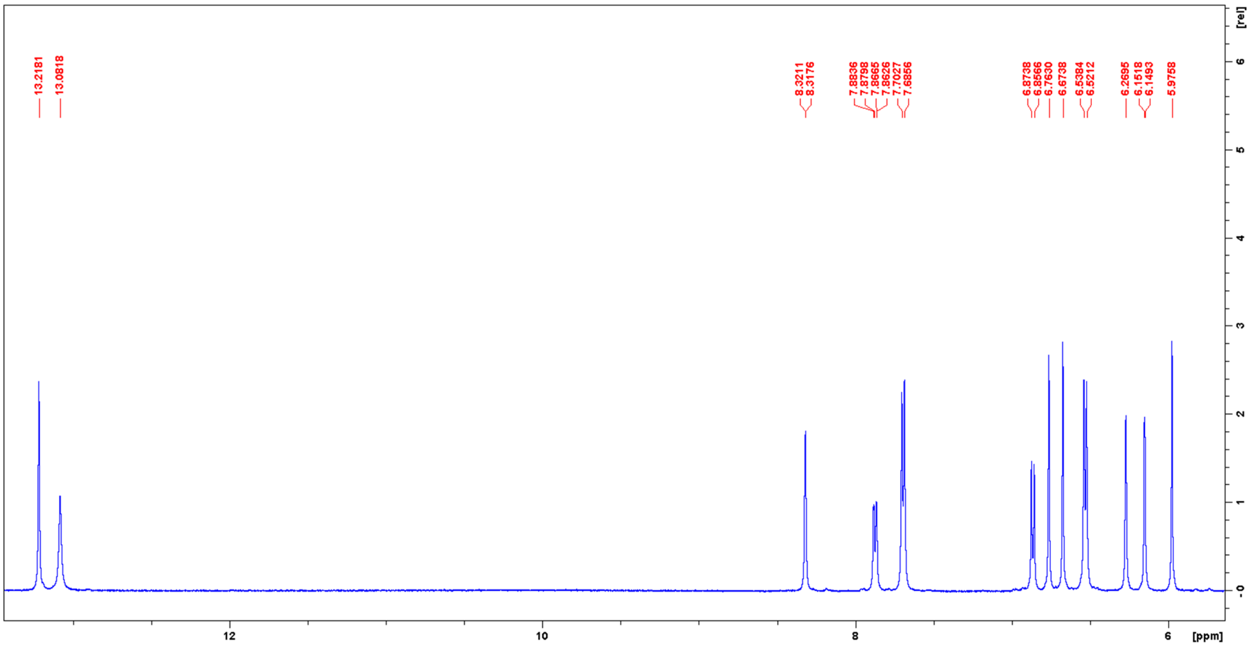

Figure S7: ^{1}HNMR of **2**.

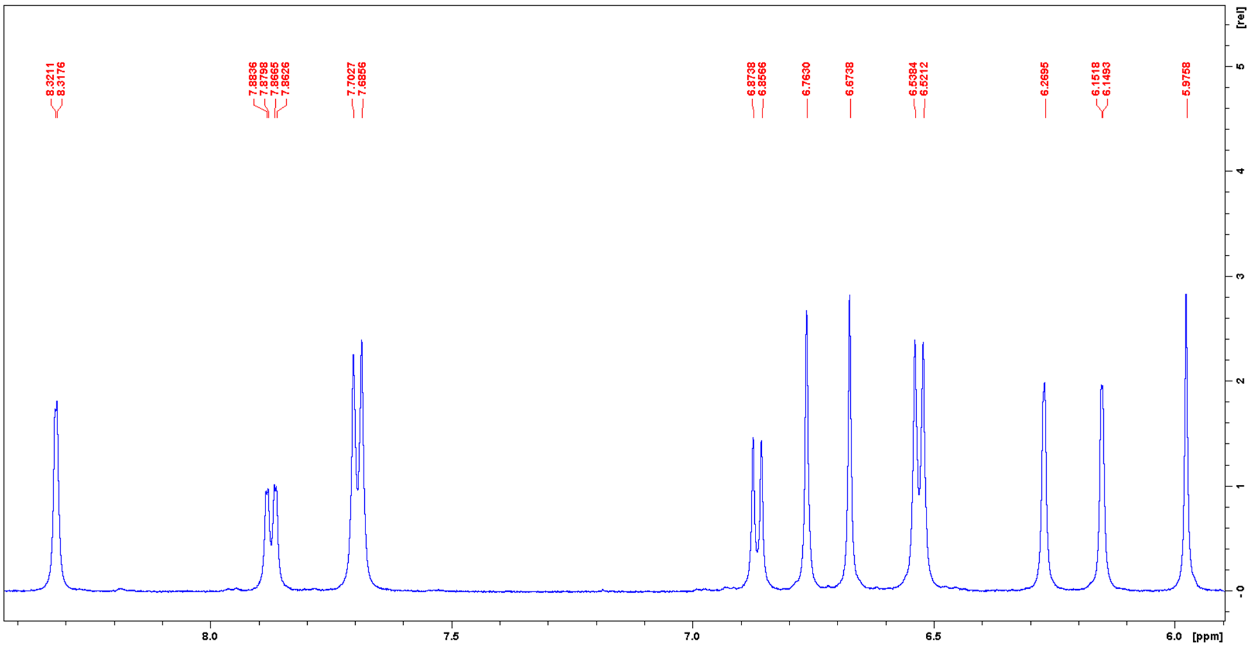

Figure S8: ^{1}HNMR of **2** (Exp.).

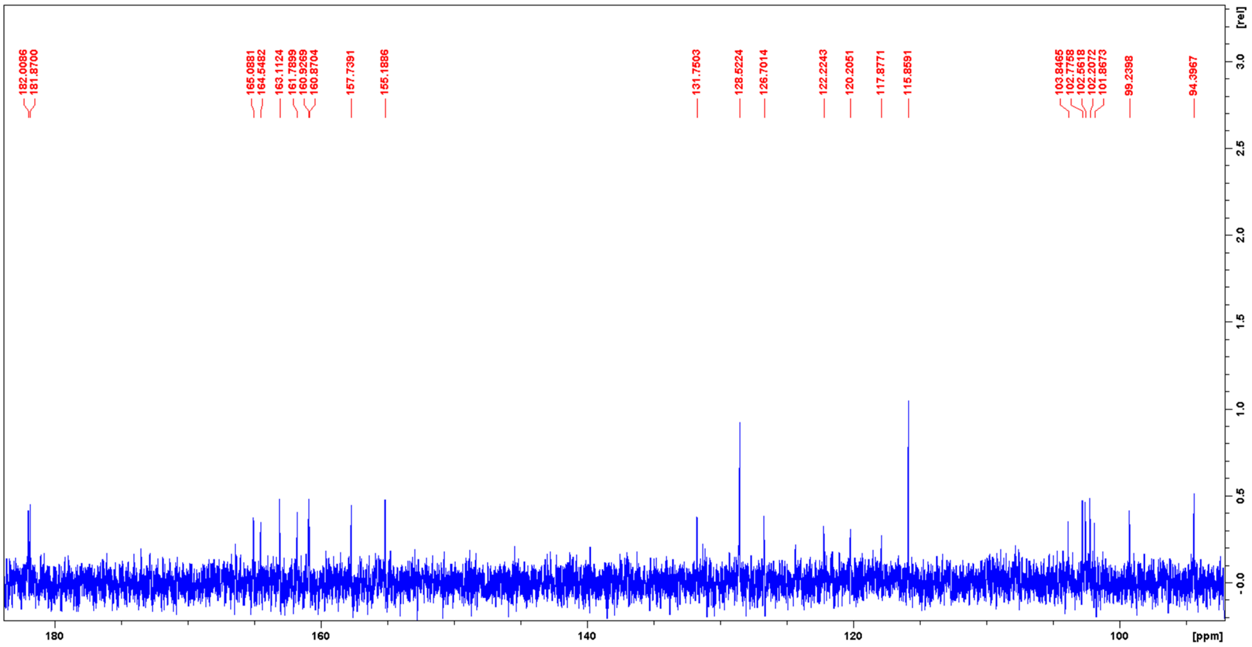

Figure S9: ^{13}CNMR of **2**.

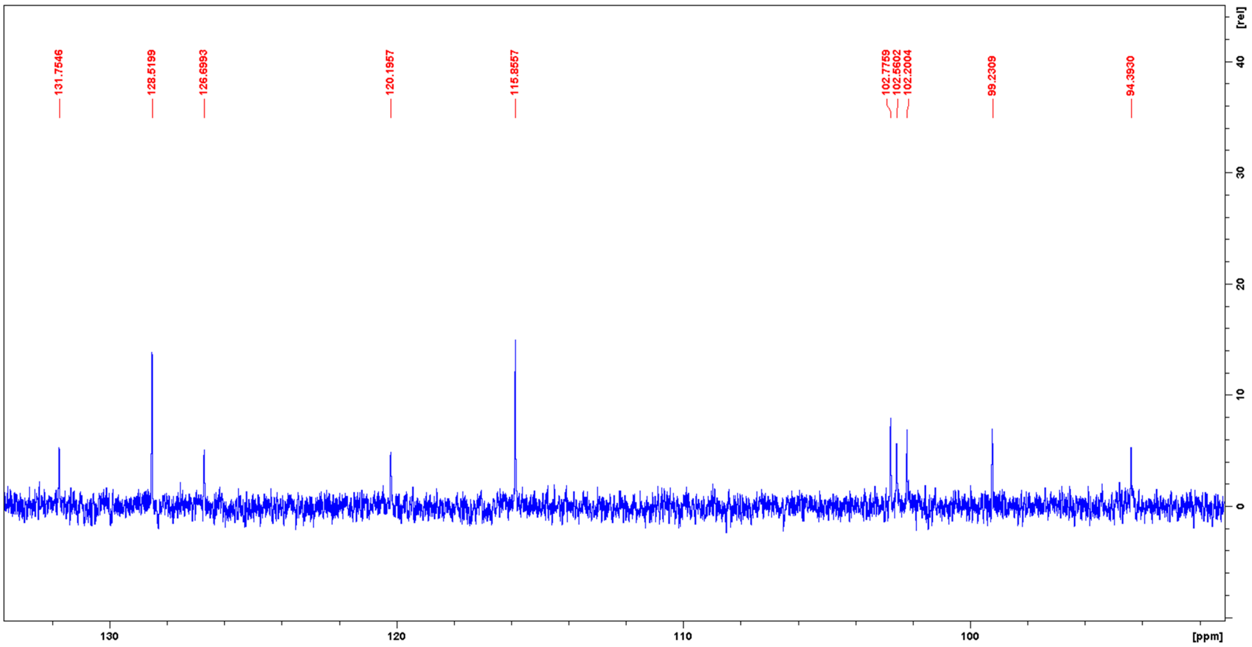

Figure S10: DEPT135 of **2**.

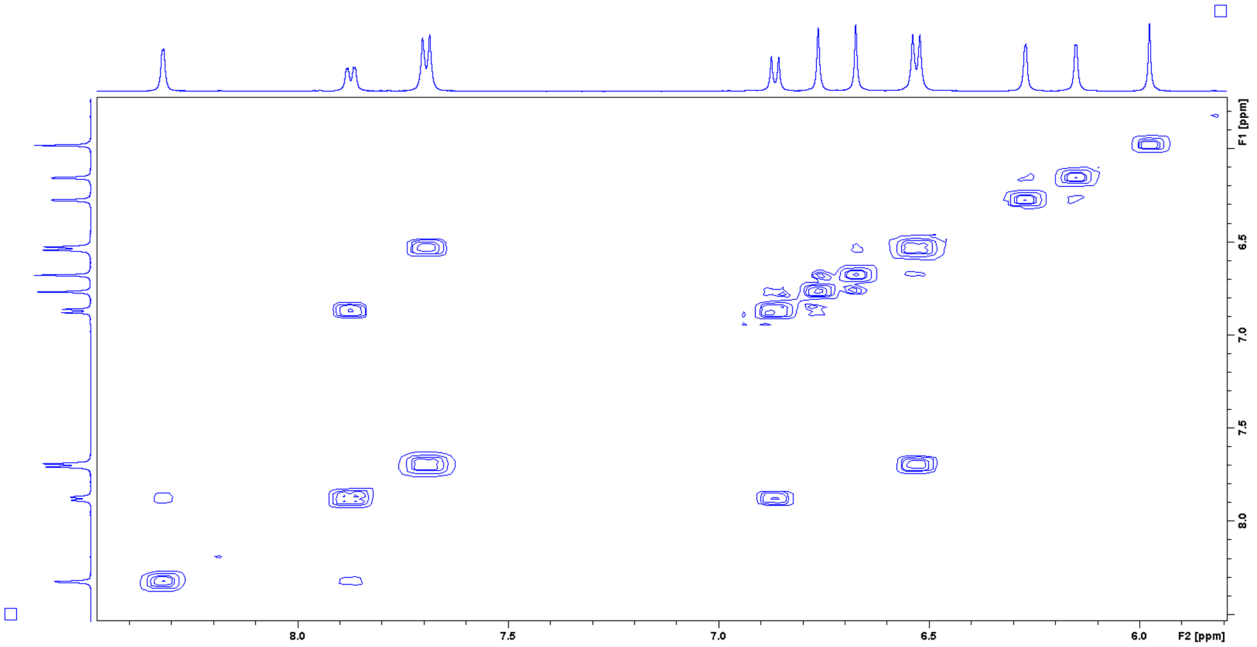

Figure S11: COSY of **2**.

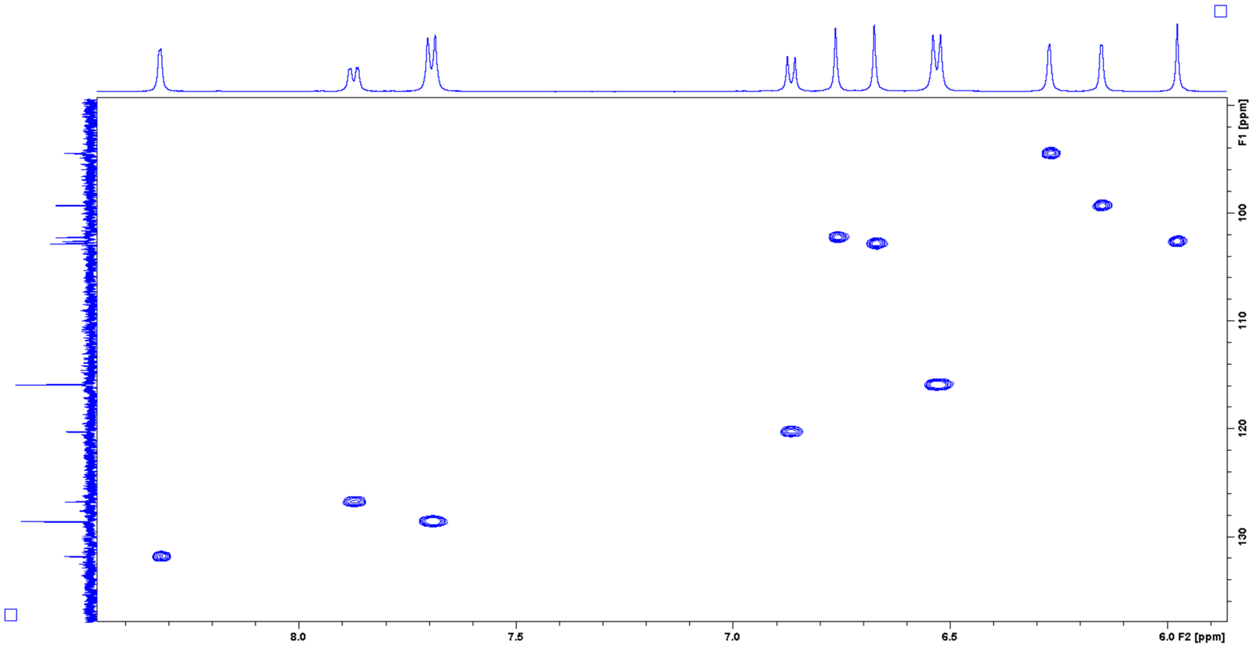

Figure S12: HSQC of **2**.

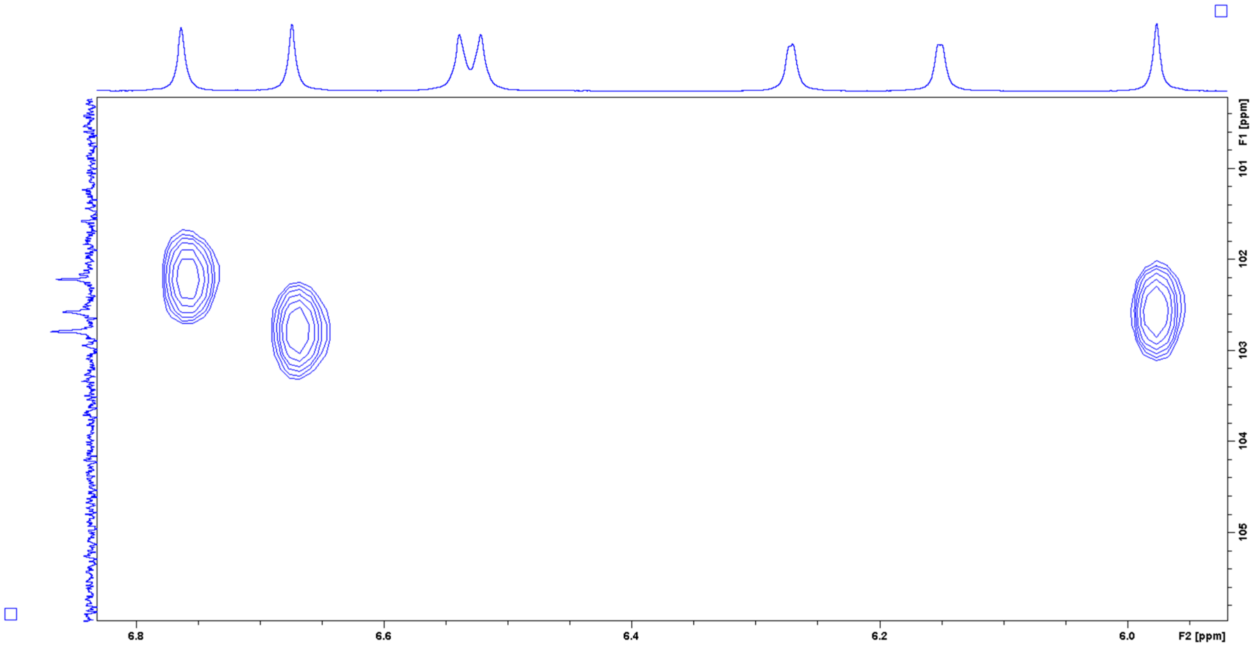

Figure S13: HSQC of **2** (Exp.).

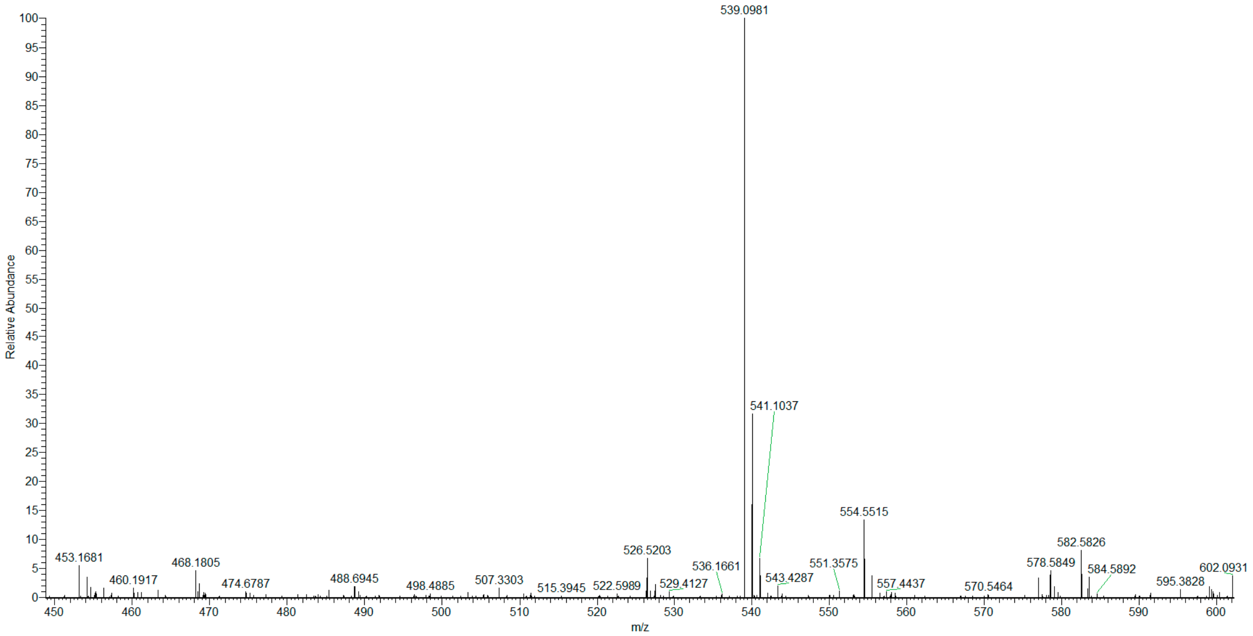

Figure S14: HRESIMS of **2**.

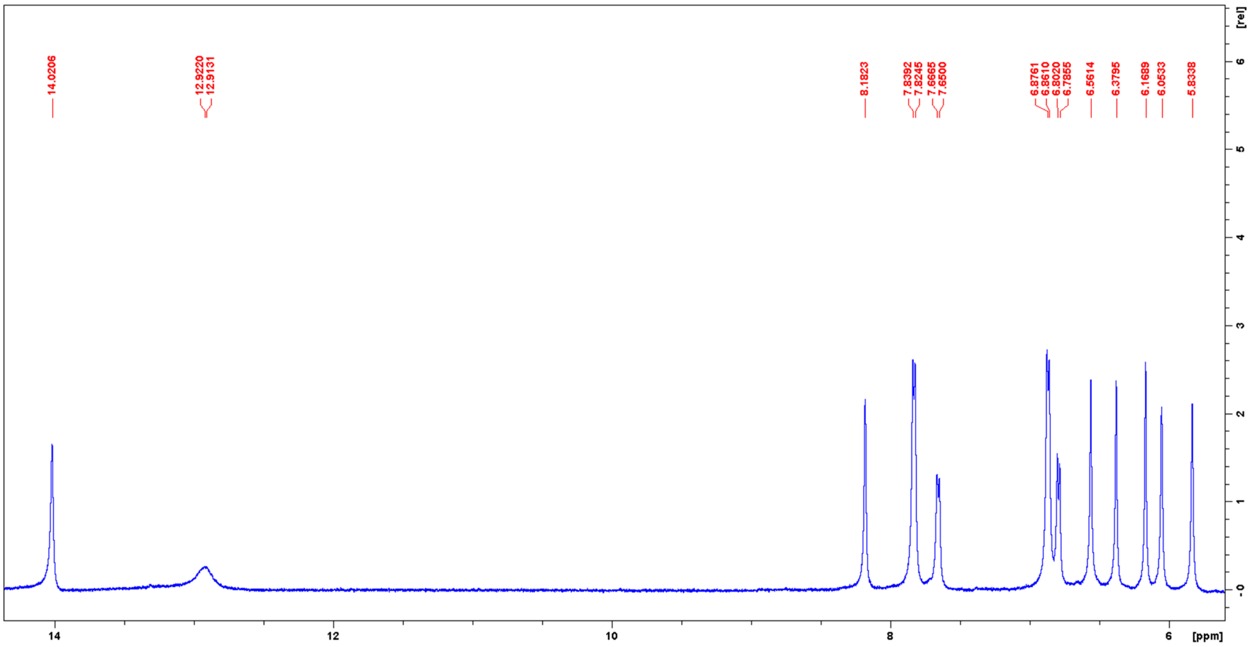

Figure S15: ^{1}HNMR of **3**.

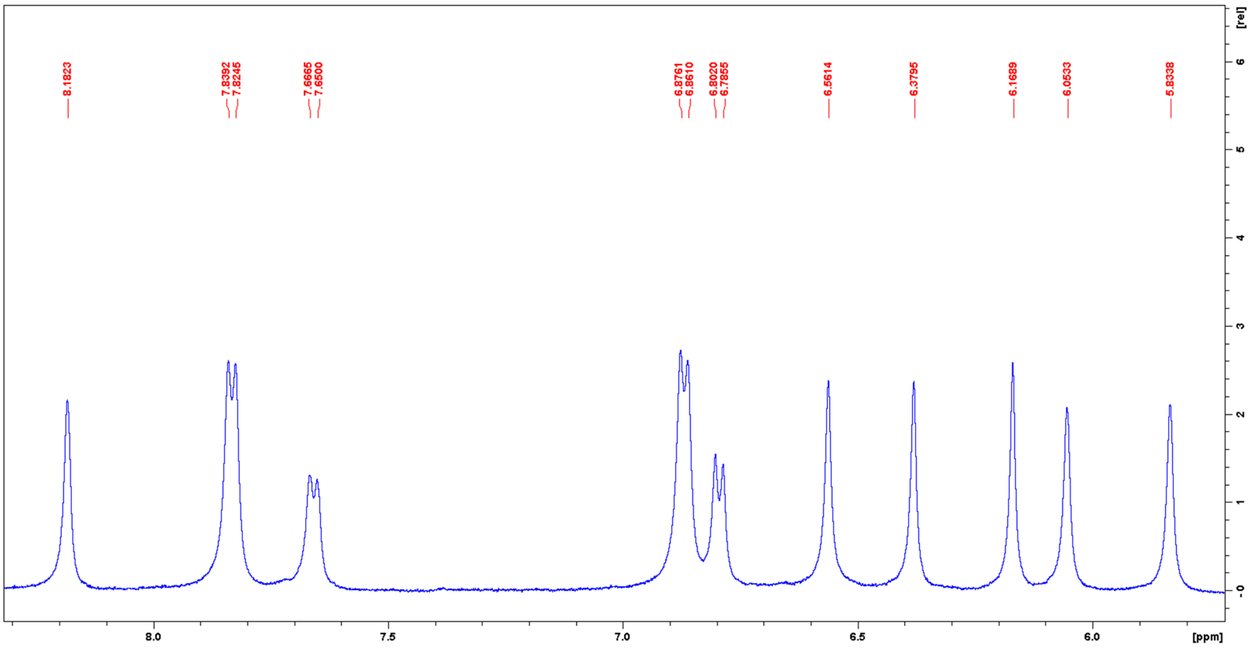

Figure S16: ^{1}HNMR of **3** (Exp.).

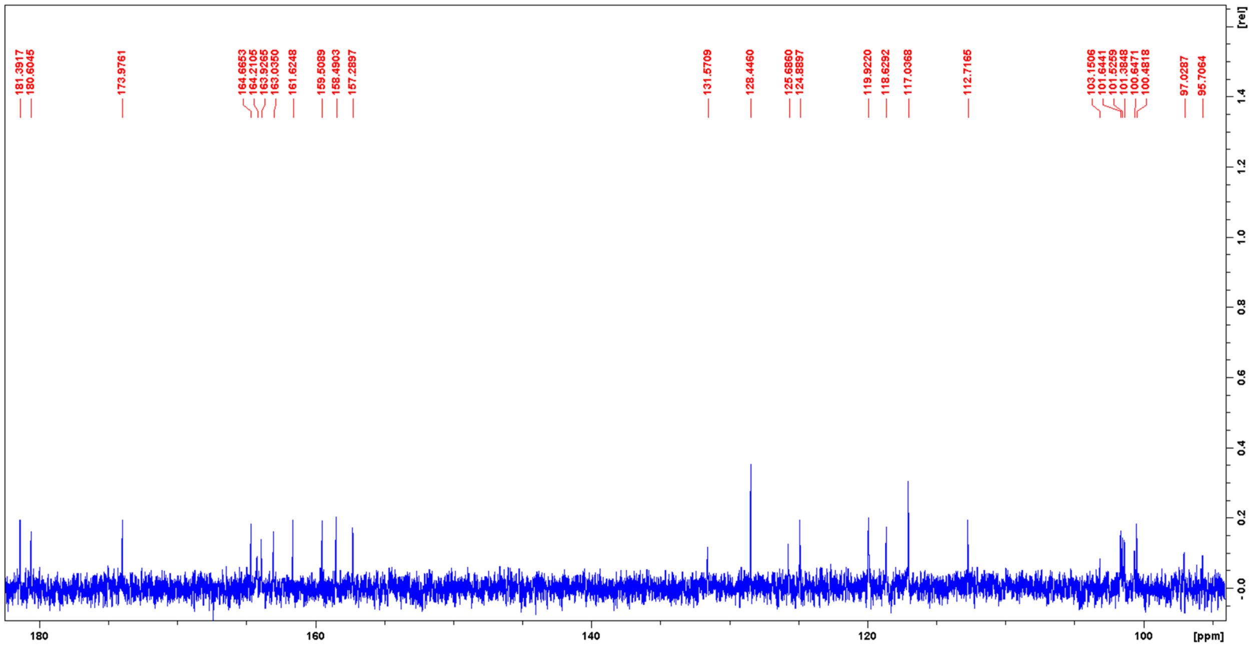

Figure S17: ^{13}CNMR of **3**.

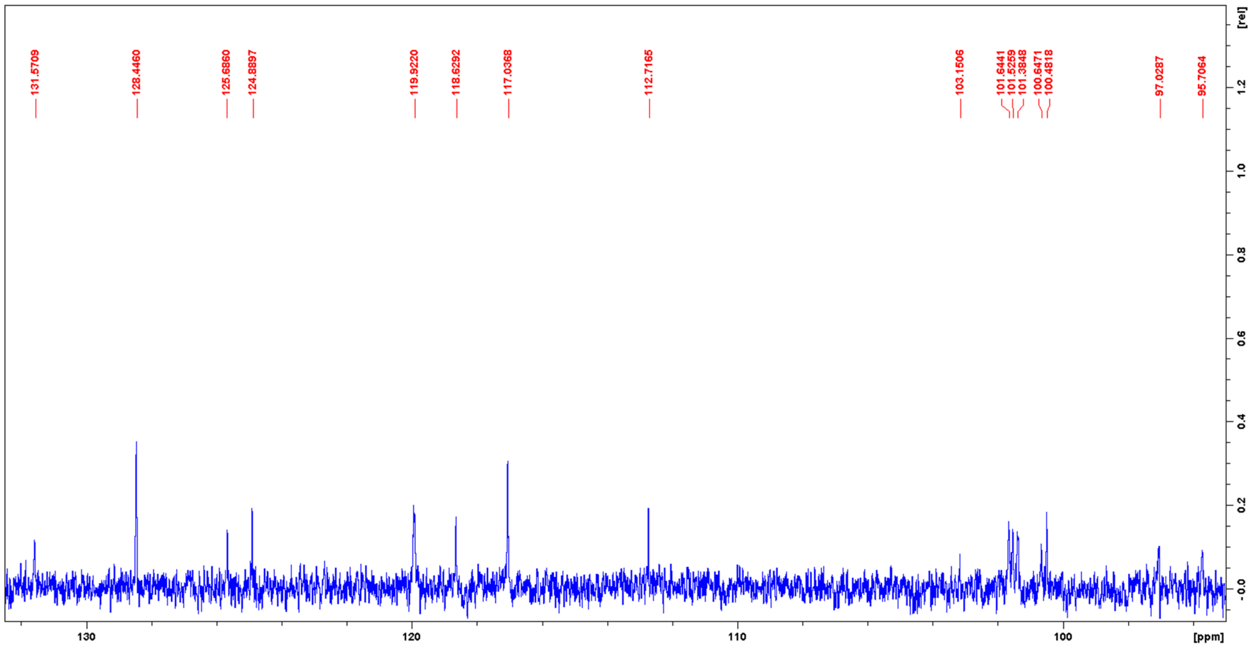

Figure S18: ^{13}CNMR of **3** (Exp.).

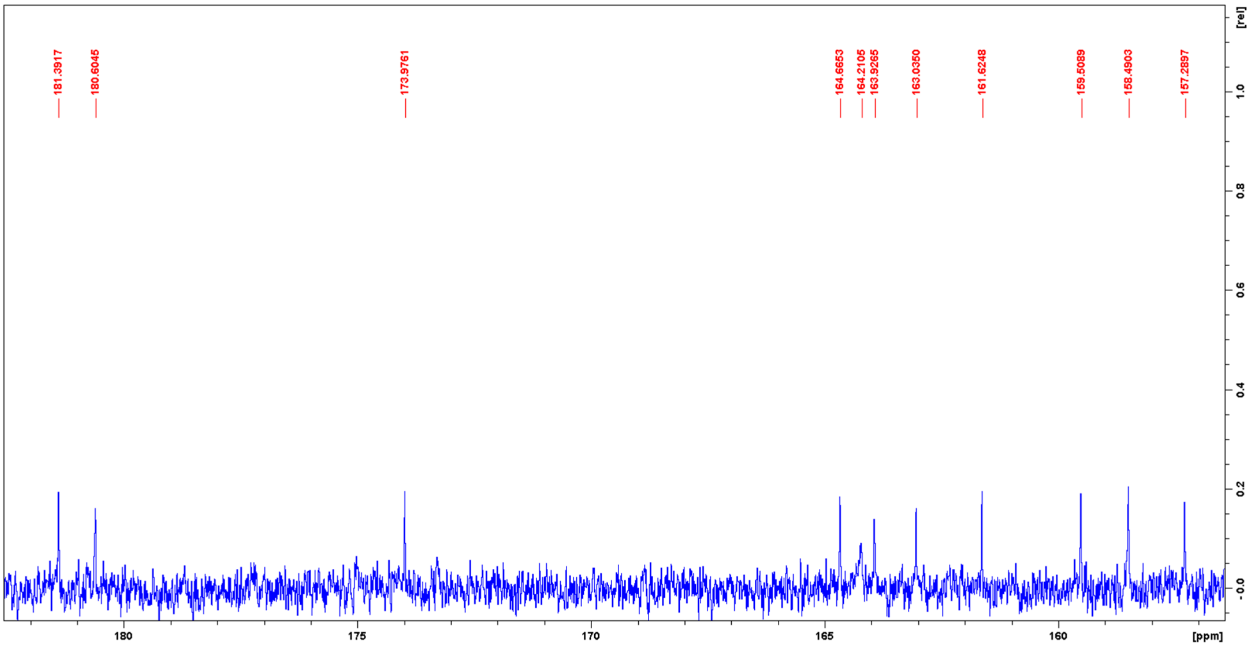

Figure S19: ^{13}CNMR of **3** (Exp.).

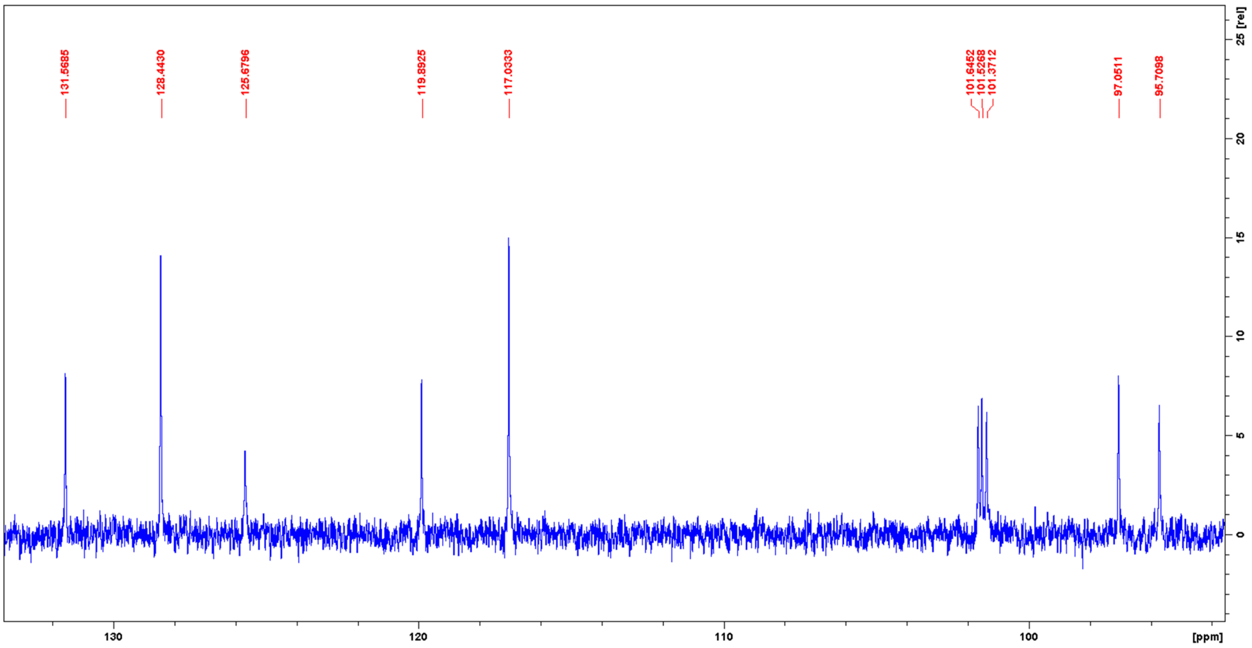

Figure S20: DEPT135 of **3**.

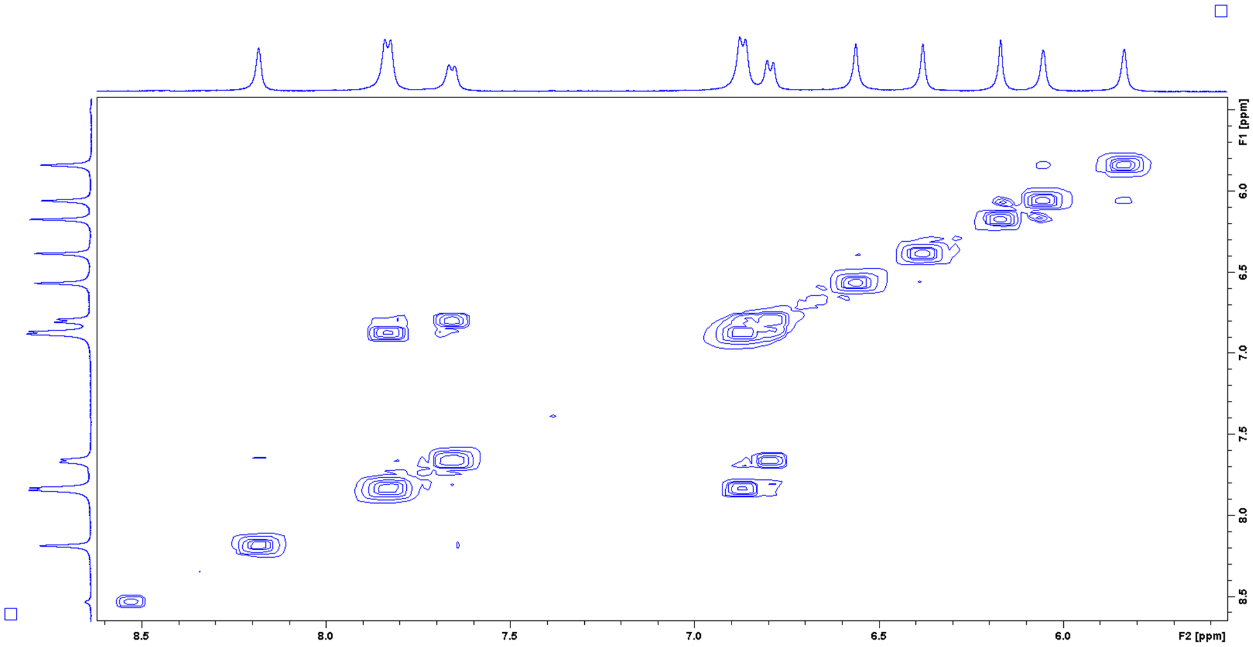

Figure S21: COSY of **3**.

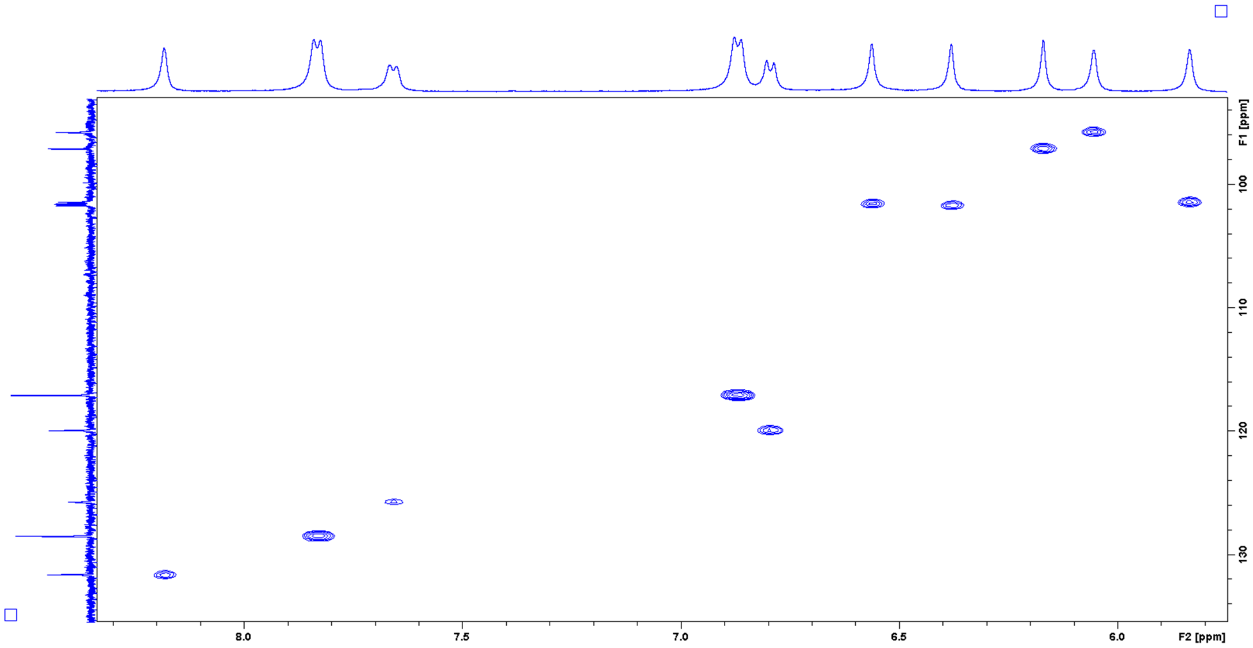

Figure S22: HSQC of **3**.

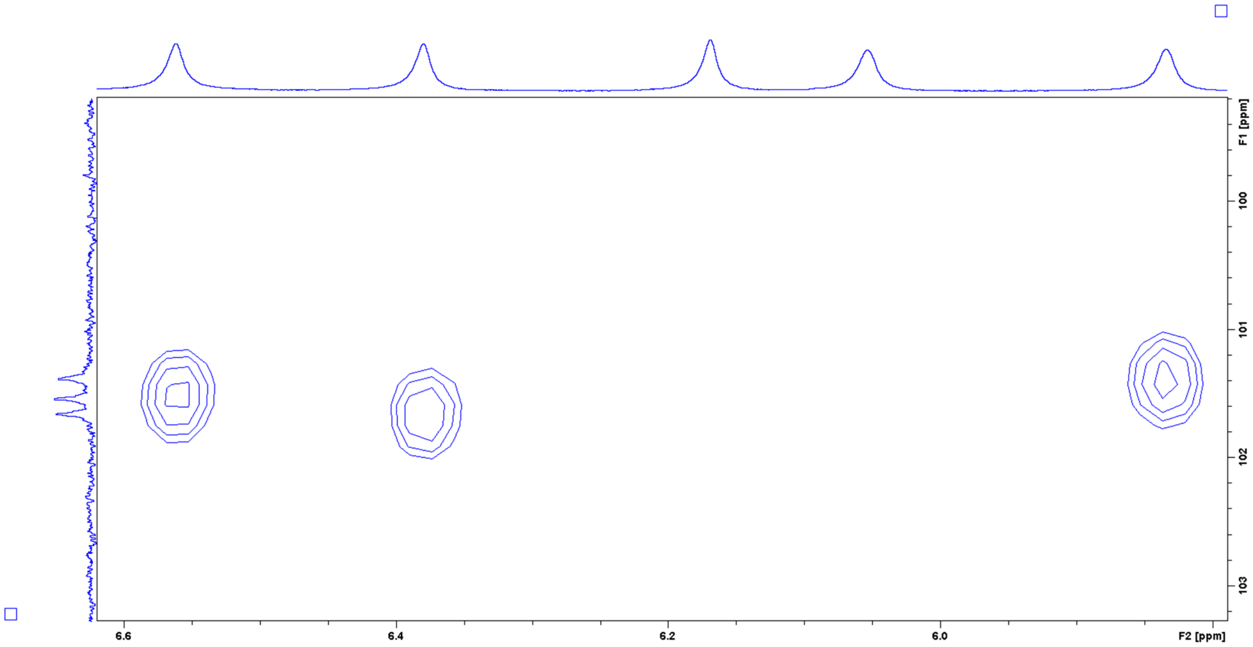

Figure S23: HSQC of **3** (Exp.).

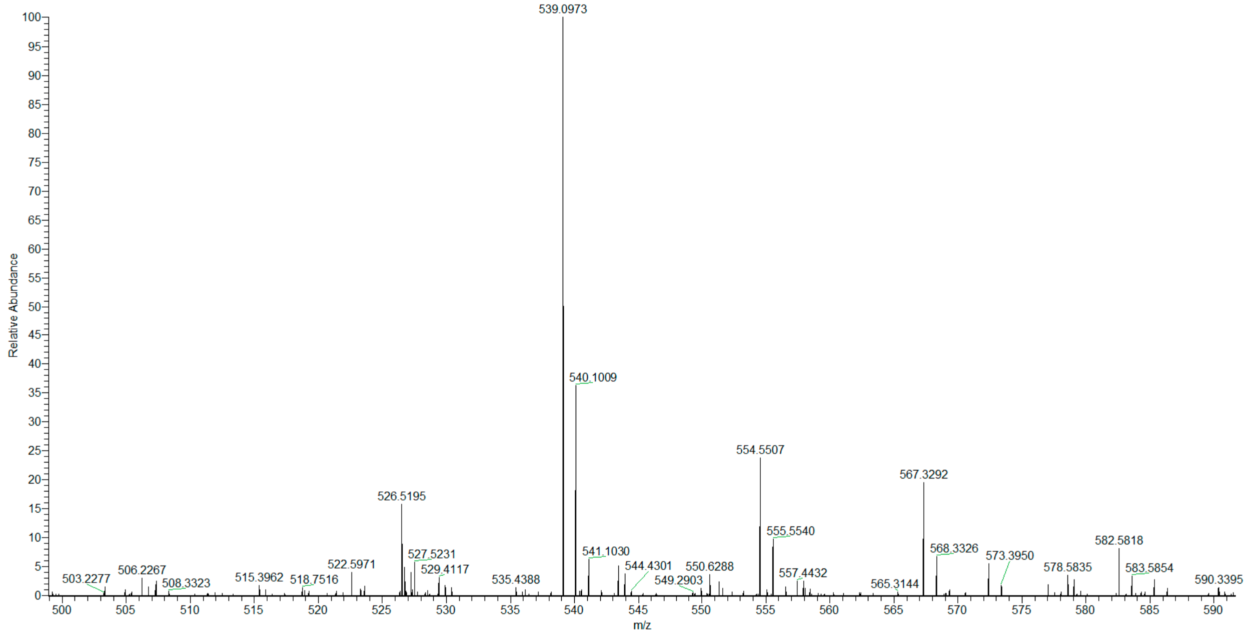

Figure S24: HRESIMS of **3**.
